# Home care for vulnerable populations with special needs during a disaster in Germany: Michael Ewers

**DOI:** 10.1093/eurpub/ckac131.061

**Published:** 2022-10-25

**Authors:** M Ewers, Y Lehmann, M Köhler

**Affiliations:** Institute of Health and Nursing Science, Charité – Universitätsmedizin Berlin, Berlin, Germany

## Abstract

**Background:**

In Europe, too, the risk of natural, technical, or man-made disasters and public health emergencies has been growing for some time. The situation of vulnerable populations with special needs receiving intensive home care such as people of all ages with oxygen therapy, peritoneal dialysis, parenteral nutrition etc. is rarely considered in this context. This issue is addressed by the sub-project “Safety and Nursing” of the AUPIK consortium on “Maintenance of home care infrastructure in crisis and disasters” funded by the German Federal Ministry of Education and Research.

**Methods:**

Starting in April 2020, a systematic literature analysis was carried out, focussing on home care of populations with special needs during disasters. This was supplemented by an online survey with nurses and care workers (n = 101) and semi-structured interviews with managers of specialized home care services (n = 8). The survey data were analysed with descriptive statistics, the interview data with content analysis; results were cross-checked with the literature.

**Results:**

Home care providers are at best prepared for everyday tasks; even minor disruptions have far-reaching consequences. Although the impact of disasters such as large-scale and prolonged power cuts are hard to imagine, the experience with the COVID-19 pandemic and other current events (e.g., floods, heat waves) could at least raise awareness of the problem. However, there is hardly any preparation for disasters in home care yet, but there are high expectations of support from civil protection organisations or local authorities. That these, in turn, are not prepared to deal with populations with special needs in intensive home care, is overlooked.

**Conclusions:**

Home care infrastructure in Germany is currently inadequately prepared in terms of concept, staff, and equipment to care for vulnerable populations with special needs during disasters. Initiatives to improve disaster preparedness in home and long-term care are overdue.

**Key messages:**

• The situation of vulnerable populations with special needs during a disaster is a pressing public health and disaster nursing issue which needs to be considered more carefully.

• Public health nursing and health services research must contribute substantially to improving disaster preparedness in all health sectors and for all populations.

